# Food industry donations to patient advocacy organisations focussed on non-communicable diseases

**DOI:** 10.1017/S1368980022001859

**Published:** 2023-03

**Authors:** Inés M Del Giudice, Krystle A Tsai, Josh Arshonsky, Sara Bond, Marie A Bragg

**Affiliations:** 1 Public Health Nutrition Program, School of Global Public Health, New York University, New York, NY, USA; 2 Department of Population Health, NYU School of Medicine, 180 Madison Ave, 3rd Floor, New York, NY 10016, USA

**Keywords:** Food industry, Funding distribution, Non-communicable diseases, Patient advocacy organisations, Conflicts of interest

## Abstract

**Objective:**

This study used publicly available Form 990 tax documents to quantify food industry donations to patient advocacy organisations (PAO) dedicated to supporting patients with non-communicable diseases.

**Design:**

Observational, cross-sectional assessment of significant national and international food industry donations to US-based non-communicable disease-focussed PAO between 2000 and 2018. Researchers recorded and categorised the: (1) frequency and value of donations; (2) reason for donation; (3) name and type of PAO recipient and (4) non-communicable disease focus of the PAO.

**Setting:**

Form 990 tax documents.

**Participants:**

Nine food and beverage companies that donated to non-communicable disease-focussed PAO.

**Results:**

Adjusting for inflation, nine food and beverage companies collectively donated $10 672 093 (*n* 2709) to the PAO between 2001 and 2018. The largest category of donations was ‘matching gifts’ (67·9 %, median amount = $115·16), followed by ‘general operations support’ (25·8 %, median amount = $107·79). Organisations focussing on cancer received the largest number and amount of donations ($6 265 861, *n* 1968). Eight of the nine companies made their largest monetary value of donation to PAO focussed on cancer.

**Conclusions:**

Publicly available tax data provide robust information on food industry donation practices. Our findings document the food industry’s role in supporting patient advocacy organisations and raise questions regarding conflicts of interest. Increased awareness of food industry donation practices involving PAO may generate pressure for policies mandating transparency or encourage donors and recipients to voluntarily disclose donations. If public disclosure becomes widespread, constituents, advocates, researchers and policymakers can better supervise and address potential conflicts of interest.

## Graphical abstract

**Figure:**
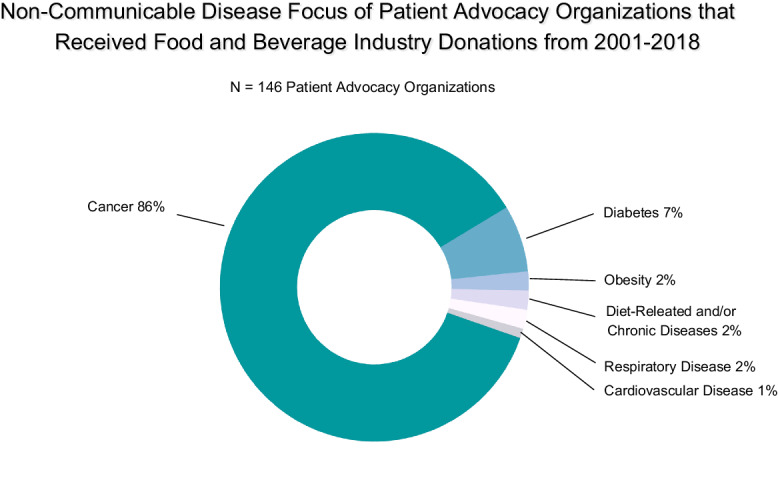

Patient advocacy organisations (PAO) are non-profits dedicated to helping patients affected by certain medical conditions^(1)^. Beyond raising public awareness about those diseases, PAO provide patient education and services and influence health policy through their lobbying activities^(1–4)^. Historically, these organisations have been praised for their support of patients. More recently, though PAO have been scrutinised due to their financial ties to different industries^(5,6)^. These financial relationships can compromise the integrity of the organisation, leading to potential conflicts of interest.

A conflict of interest occurs when ‘an institution’s own financial interests or the interests of its senior officials pose risks to the integrity of the institution’s primary interests and missions’^(7)^. Conflicts of interest can emerge when advocacy organisations receive funding from companies that promote products or services that may be at odds with the mission of the advocacy organisation. In 2015, for example, the New York Times revealed that in the previous year, Coca-Cola had donated $1·5 million to start the Global Energy Balance Network—a non-profit that minimised the role of diet as a driver of obesity and instead overemphasised the role of physical inactivity^(8)^.

Past scholarship has documented extensive conflicts of interest within the tobacco and pharmaceutical industries,^(9–11)^ but few studies investigate food industry donations to PAO, specifically. Research sponsored by the food industry has been shown to support industry aims^(12–20)^. And relationships between the food industry and academia have been shown to influence medical journalism^(20)^ and public policy^(21)^. In 2011, for example, the American Beverage Association donated over $10 million for ‘childhood obesity prevention initiatives’ to the Children’s Hospital of Philadelphia^(22)^. The association happened to donate this generous amount just as the City Council was deliberating over a soda tax proposal. In the end, the Council rejected the tax, showing the extent to which industry can influence academic programmes and public health policies. Public health experts question the motivations behind food industry donations such as the one in Philadelphia, and worry that food corporations presenting themselves as part of the solution to obesity and other diet-related health problems may actually undermine efforts to enact meaningful public health policy. Corporations are, by definition, obligated to sell products—even when such products are at odds with promoting good health. They must be able to make a profit, and as history shows, they are willing to interfere with public health policies that may jeopardise those profits.

External financial support is valuable for PAO, especially because they tend to rely on industry donations to fund their work^(1,23)^. A 2013 and 2014 survey conducted by researchers at Case Western Reserve University of PAO leaders in the USA revealed that 67 % of PAO reported receiving private industry funding at a median amount of $50 000 in their prior fiscal year^(6)^. Recent studies from the Perelman School of Medicine in Philadelphia, Pennsylvania also document financial support from the pharmaceutical, device and/or biotechnology industries to PAO^(2,6,24,25)^. In a study by McCoy *et al.*, authors found that 83 % of PAO received financial support from partners in the pharmaceutical industry. The majority of those PAO (88 %; *n* 104) published a list of donors, but only 57 % published the amount of donations they received^(2)^. To increase transparency of financial relationships between PAO and the pharmaceutical industry, Kaiser Health News developed the PreScription for Power database. Researchers from PreScription for Power tracked $162·6 million donated to 650 PAO by twenty-six pharmaceutical companies in 2015^(24)^. To our knowledge, however, no studies have examined the extent to which food and drink companies fund PAO dedicated to fighting non-communicable diseases (i.e. CVD, cancer and diabetes). Given the links between sugar-sweetened beverage and ultra-processed food intake and non-communicable diseases (e.g. diabetes)^(26–29)^, documenting food industry funding to PAO focussed on non-communicable diseases is a critical first step in increasing transparency addressing potential conflicts of interest^(30)^.

To address this gap in research, the present study, conducted in New York City, aimed to quantify the frequency and types of national and international food industry donations to PAO focussed on non-communicable diseases in the USA over an 18-year period. We aimed to: (1) document the frequency and monetary value of donations; (2) identify any stated reasons for donations; (3) quantify the percentage of funding distributed among chronic health conditions and (4) quantify the percentage of money per health condition per food company.

## Methods

We conducted an observational, cross-sectional assessment of significant national and international food industry donations to US-based non-communicable disease-focussed PAO between 2000 and 2018. The Institutional Review Board at New York University School of Medicine exempted this study from review.

### Sample

We defined non-communicable disease-focussed PAO as non-profit groups whose primary mission is to combat a non-communicable disease or improve the health and well-being of a patient population^(2)^. In our study, we focussed on PAO tackling diet-related non-communicable diseases, including the most prevalent ones: CVD (heart disease and stroke), cancer, chronic respiratory diseases, diabetes and obesity^(31)^.

We defined the *food industry* as any company whose primary objective is to sell food or beverage products^(30)^. To identify food and beverage companies, we used the Food Advertising to Children and Teens Scores (FACTS) reports^(32–36)^ published by the Rudd Center for Food Policy and Obesity. These reports rank companies based on their marketing budgets in six categories: fast food, sugary drink, children’s drinks, baby food, snack food and cereals. Research assistants identified 101 companies within the categories most relevant to adults: fast food, sugary drinks, cereals and snacks. We excluded companies in the categories of ‘children’s drinks’ and ‘baby food’ in order to conduct a future study on a number of comprehensive issues relevant to younger age groups, including physical health, but also other factors related to child development.

**Fig. 1:**
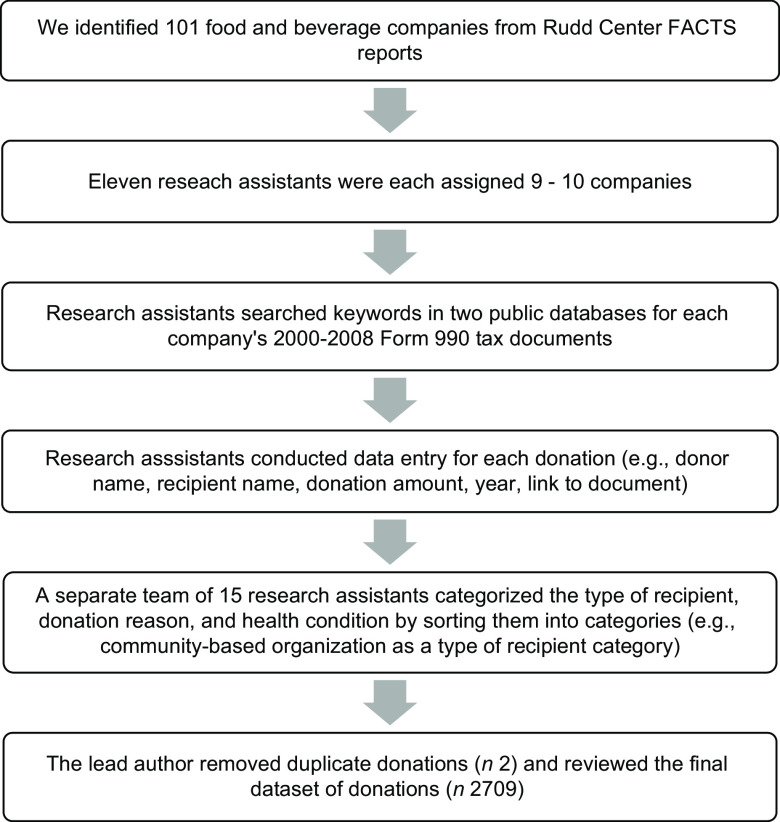
Flow chart of the online search processes and data collection, coding and cleaning

In 2020, we randomly assigned nine or ten of the 101 identified food and beverage companies to eleven research assistants, assigning no single company to more than one assistant. We trained the research assistants to search for donations from their assigned companies between 2000 and 2018 using the procedures described in Fig. 1. Researchers used independent investigative journalism site www.propublica.org and non-profit search database www.guidestar.org to identify donations using every combination of the following keywords: name of the assigned food or beverage company plus the words: ‘foundation’, ‘contribution’, ‘donation’, ‘gift’, ‘funding’, ‘grant’ or ‘financial support’. These searches yielded results that included food or beverage company websites, media press releases and US Form 990 filings. Form 990 is a US Internal Revenue Service document that all tax-exempt organisations (e.g. The Coca-Cola Foundation, Inc.) are required to file annually. This document provides information about the organisation to the Internal Revenue Service, promotes tax compliance and assists the government with charitable and regulatory oversight. Form 990 is also open to public inspection and allows organisations to share information about their programmes with the public. We asked research assistants to download and save only the tax forms covering the 18-year period. These documents include information on the corporate sponsor, donor recipients, the nature of the support, the year the donation was distributed and the monetary value of the donation.

Based on our previously described methodology, we instructed researchers to limit their donation searches to 5 h/company, unless they continued to find additional donations^(30)^. After excluding sixty-eight companies for which they could not find any donations, the research assistants collected data for thirty-three companies. After collecting the data, thirteen companies were excluded due to a lack of donations to non-communicable disease-focussed PAO. Eleven companies were excluded due to lack of complete data on donations from 2000 to 2018; those included Burger King, Essentia Water, General Mills Foundation, Jack in the Box, Mars, McDonald’s, PepsiCo, Quaker, Spindrift, Kellogg and Yum! Brands. Our final sample included the following nine food and beverage companies: Clif Bar & Company, The Coca-Cola Company, Newman’s Own, Mondelez International, Wendy’s, The Kraft-Heinz, Ferrero USA Inc, Campbell’s Soup and Chick-fil-A. We excluded the year 2000, as tax documentation from our selected food companies could not be found.

### Data collection and analysis

A separate team of fifteen research assistants then examined the 2001–2018 tax documents collected by the previous set of researchers and collectively spent 46 h recording and organising the data into: (1) the frequency and value of donations; (2) reason for the donation; (3) the name and type of PAO recipient and (4) the non-communicable disease focus, if any, of the PAO. Research assistants categorised PAO using keywords that appeared in their mission statements online. These included a combination of the following nine non-communicable disease keywords: ‘obesity’; ‘heart’; ‘CVD’; ‘cancer’; ‘tumor’; ‘lung disease’; ‘asthma’; ‘chronic disease’; or ‘diet-related’, and 12 advocacy keywords: ‘prevent’; ‘cure’; ‘fight’; ‘advocacy’; ‘education’; ‘research’; ‘raise money’; ‘fund’; ‘awareness’; ‘improve lives’; ‘save lives’ or ‘support’.

Using the descriptions of each donation in the companies’ tax forms, we categorised the reasons for donating into eight categories: (1) research; (2) educational initiative; (3) miscellaneous programme (i.e. family support programme or building stronger communities); (4) matching gift (i.e. when a company matches the amount its employees donate to a non-profit organisation); (5) general operations support; (6) scholarship and fellowship; (7) health and human services and (8) environmental initiative. Using mission statements, we organised health conditions into the following categories: (1) CVD; (2) cancer; (3) respiratory disease; (4) diabetes; (5) obesity and (6) multiple diseases and/or singular diet-related/chronic diseases that are not specified in one of the previous categories (e.g. chronic kidney disease).

After research assistants finished recording and coding data, the lead author searched for and removed duplicate donations (*n* 2) and cleaned the final dataset of donations (*n* 2709). To verify reliability in the coding process, a separate team of three research assistants re-coded 1500 donations from the dataset. The lead author then calculated the percentage of agreement, ensuring that agreement for all variables was above 90 %. We adjusted the donation amounts for inflation by calibrating them to the year 2018—the final year of our data collection period—using the World Bank’s US historical inflation rates^(37)^. We then quantified the frequency and monetary value of donations for each company and for the entire sample. We also calculated the frequencies of donation reasons listed, as well as health conditions targeted. Finally, we calculated the total number, monetary value and percentage of donations per health condition per company.

## Results

### Number and monetary value of donations over time

**Fig. 2:**
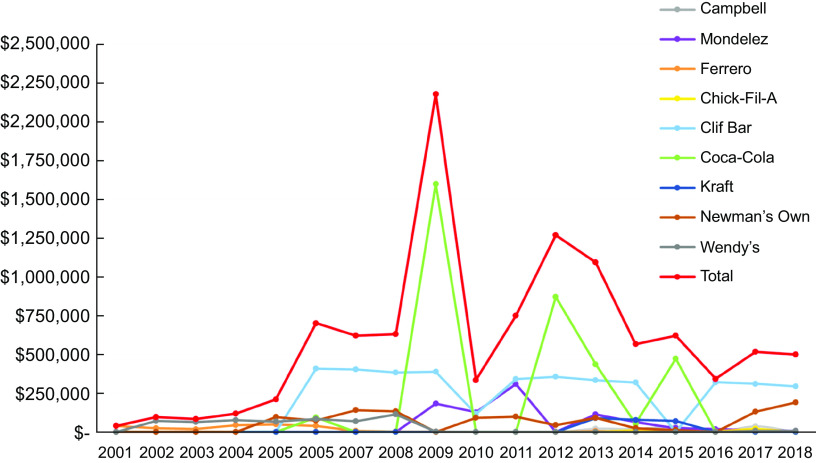
Trends in food and beverage company donations made to non-communicable disease-focussed PAO from 2001 to 2018

The food and beverage companies in our sample collectively made 2709 times donations to 146 PAO in our sample between 2001 and 2018 (Fig. 2). Between 2001 and 2009, the total annual monetary value of donations increased from $38 800 (*n* 4) to $2·2 million (*n* 307). From 2009 to 2010, the total monetary value of donations declined from $2·2 million to $335 000, but the number of donations stayed almost the same (*n* 309). From 2010 to 2018, the number and monetary value fluctuated based on the data provided on the tax forms, with the largest monetary value occurring in 2012 ($1·3 million, *n* 18). The highest number of donations occurred in 2013 (*n* 555).

### Monetary value of the donations and years the donations were distributed

Table 1 lists the donors, amounts and number of donations, and the number of years for which we found donations for a given company. In total, the nine companies in our sample donated $10·7 million, adjusted for inflation. Clif Bar & Company was the largest donor; its seventy donations totalled nearly $4 million and accounted for 36·9 % of the total monetary value of donations we studied.

**Table 1:** Summary of public information on nine food company donations to non-communicable disease-focussed PAO between 2001 and 2018

Name of donor company	Total donations amount, adjusted for inflation	Median donation value/year, adjusted for inflation	Total number of donations	Number of years actively donating	Median number of donations/year
Clif Bar & Company (e.g. snack bars)	$3 976 003	$337 596	70	12	5·5
The Coca-Cola Company (e.g. sugary drinks)	$3 521 460	$453 917	14	6	2·5
Newman’s Own (e.g. salad dressings)	$1 131 954	$94 281	90	12	8
Mondelez International Inc (e.g. chocolate bars)	$857 327	$63 070	1798	9	204
Wendy’s (e.g. fast food)	$560 902	$67 322	33	10	3·5
The Kraft-Heinz Company (e.g. condiments)	$237 591	$77 845	655	3	219
Ferrero USA, Inc. (e.g. chocolate-hazelnut spreads)	$233 527	$12 696	29	12	3
Campbell’s Soup Company (e.g. canned soups)	$114 320	$28 265	14	4	1
Chick-fil-A (e.g. fast food)	$39 008	$7952	6	4	1
Total	$10 672 093	$77 845	2709	x˜: 9	x˜: 3·5

Table 2 lists the ten largest individual donations in our sample. In 2009, the Coca-Cola Company made the largest individual donation ($1 million) to support operations of the British Nutrition Foundation, whose mission is to translate ‘evidence-based nutrition science in engaging and actionable ways’^(38)^. The Coca-Cola Company gave the second largest individual donation ($436 000 made in 2013 to EPODE International Network, an organisation that aims to prevent childhood obesity^(39)^.

**Table 2:** Ten largest donations, adjusted for inflation, to non-communicable disease-focussed PAO by food companies between 2001 and 2018, ranked by total monetary amount

Donor company	Name of recipient	Year	Donation amount	Reason for donation	Language from data source	Health condition
The Coca-Cola Company	British Nutrition Foundation	2009	$1 041 709	General operations support	“Foundation Contribution”	Diet-Related Diseases and/or Chronic Diseases
The Coca-Cola Company	EPODE International Network	2013	$436 554	General operations support	“EPODE France”	Obesity
The Coca-Cola Company	Magyar Dietetikusok Orszagos Szovetsege (Hungarian Dietetic Association)	2012	$366 390	Scholarship and fellowship	“Dietitian Support Program for Hungarian University Students”	Diet-Related Diseases and/or Chronic Diseases
The Coca-Cola Company	French Diabetics’ Association	2012	$328 110	Health and human services	“Balanced Diet and Physical Activity for Diabetic Peer Support Groups”	Diabetes
Clif Bar & Company	Breast Cancer Fund	2014	$307 604	Environmental initiative	“Reducing environmental health hazards”	Cancer
Clif Bar & Company	Breast Cancer Fund	2008	$334 034	General operations support	“Charitable”	Cancer
Clif Bar & Company	Breast Cancer Fund	2007	$345 156	General operations support	“Charitable”	Cancer
Clif Bar & Company	Breast Cancer Fund	2006	$356 995	General operations support	“Charitable”	Cancer
The Coca-Cola Company	American Council for Fitness and Nutrition Foundation	2009	$321 876	General operations support	“Foundation Contribution”	Obesity
The Coca-Cola Company	Fondazione Diabete Ricerca Onlus – Diabetes Research Foundation	2015	$217 187	Research	“Epode Umbria Region Obesity Intervention Study”	Diabetes

### Purpose of donations as categorised based on information presented in company tax reports

Research assistants identified a reason for 94 % of the donations (*n* 2557). The identified reasons represented eight broad categories that were not mutually exclusive (i.e. some donations listed more than one reason) (Table 3). The largest categories included ‘matching gifts’ (67·9 %, *n* 1738, median amount = $115·16); ‘general operations support’ (25·8 %, *n* 661, median amount = $107·79); ‘miscellaneous programs’ (3·2 %, *n* 81, median amount = $8164·08) and ‘research’ (1·8 %, *n* 46, median amount = $4754·74).

**Table 3:** Purpose of donations as categorised based on information presented in food and drink company tax reports from 2001 to 2018

Reason for donation	Number (%) of donations with that reason listed (*n* 2557)	
	*n*	%
Matching gifts	1738	67·9
General operations support	661	25·8
Miscellaneous programmes	81	3·2
Research	46	1·8
Health and human services	10	0·4
Environmental initiative	10	0·4
Educational initiatives	8	0·3
Scholarship and fellowship	5	0·2
Total number of reasons listed*	2559	

* There were 2557 donations with one or two specific reasons for the gift. As some donations had more than one reason listed, the total is 2559.

### Number and monetary value of the donations to each health condition

Compared to other non-communicable diseases, cancer received the largest number and amount of donations from the food industry ($6·26 million, *n* 1968) (Table 4). CVD received the second largest number of donations (*n* 364) but ranked fifth in total monetary value ($357 000), followed by respiratory disease ($82 000). Finally, diabetes ranked third in both number and amount of donations ($1·37 million, *n* 315).

**Table 4:** List of health conditions that received donations from nine food companies from 2001 to 2018, ranked by total number of donations

Health condition	Number (%) of donations to that health condition listed		Total donation amount (%) to that health condition, adjusted for inflation	
	*n*	%	*n*	%
Cancer	1968	72·6	$6 265 861	58·7
CVD	364	13·4	$356 733	3·3
Diabetes	315	11·6	$1 371 638	12·9
Respiratory disease	54	2	$82 247	0·8
Diet-related diseases and/or chronic diseases	4	0·1	$1 636 063	15·3
Obesity	4	0·1	$959 552	9
Total	2709		$10 672 093	

### Number and monetary value of donations from nine food companies to each health condition

Table 5 lists food company name, number, monetary value and percentage of donations to each health condition. Eight of the nine companies included in the analysis made their largest monetary value of donation to PAO focussed on cancer. The only exception was Coca-Cola, which made the largest donation to PAO focussed on ‘diet-related diseases’ and/or ‘chronic diseases’.

**Table 5:** Food company donations to health conditions from 2001 to 2018

Company name	Health condition	Number of donations	Total donation amount (%), adjusted for inflation	
			*n*	%
Clif Bar & Company	Cancer	51	$3 841 842	96·6
	Diabetes	19	$134 161	3·4
The Coca-Cola Company	Cancer	2	$93 422	2·7
	Diabetes	4	$832 424	23·6
	Obesity	4	$959 552	27·2
	Diet-related diseases and/or chronic diseases	4	$1 636 063	46·5
Newman’s Own	Cancer	62	$847 134	74·8
	Diabetes	11	$96 587	8·5
	CVD	16	$175 777	15·5
	Respiratory disease	1	$12 456	1·1
Mondelez International, Inc	Cancer	1335	$635 490	74·1
	Diabetes	206	$147 475	17·2
	CVD	216	$70 392	8·2
	Respiratory disease	41	$3970	0·5
Wendy’s	Cancer	14		
	Diabetes	6	$130 447	23·3
	CVD	7	$78 330	14
	Respiratory disease	6	$64 974	11·6
The Kraft Heinz Company	Cancer	459	$177 993	74·9
	Diabetes	68	$28 212	11·9
	CVD	122	$30 539	12·9
	Respiratory disease	6	$846	0·4
Ferrero USA	Cancer	26	$229 590	98·3
	Diabetes	1	$2333	1
	CVD	2	$1604	0·7
Chick-fil-A	Cancer	6	$39 008	100
Campbell Soup Company	Cancer	13	$114 230	99·9
	CVD	1	$90	0·1

## Discussion

This investigation generated the largest database to date of food industry donations to non-communicable disease-focussed PAO. The data show the extent of food industry donations to organisations and raise questions about potential conflicts of interest that may arise. The three reasons provided most frequently for food company’s donations included matching gifts, general operations support and miscellaneous programmes. Although food and beverage company support may enable PAO to engage in valuable research, education and advocacy activities, it is also possible that these relationships may result in conflicts of interest. Case in point: many academic institutions and universities have received gift donations from opioid companies, often using these large gifts to establish research centres and degree programmes. Thousands of documents made public in 2019 revealed how Purdue Pharma’s relationships with academic institutions provided them with opportunities to influence research, curricula, speaker series and other events^(10)^. Companies in the pharmaceutical industry—as well as those in other industries—understand how non-profit organisations depend on and are profoundly influenced by their gifts and relationships. And they regularly exploit this relationship with organisations to promote policies that protect their interests. In a working paper from 2018, the National Bureau of Economic Research concluded that ‘corporations strategically deploy charitable grants to induce non-profit grantees to make comments that favor their benefactors, and that this translates into regulatory discussion that is closer to the [corporation’s] own comments’^(40)^.

Previous research showed that Coca-Cola and PepsiCo sponsored a total of ninety-six national health organisations between 2011 and 2015, including the Academy of Nutrition and Dietetics^(41)^. Six of these ninety-six organisations are non-communicable disease-focussed PAO organisations that received donations from Coca-Cola in our sample. Nearly three-quarters of the total number of donations, and more than half of total monetary value, was made to cancer-focussed PAO, supporting previous research in funding distributions that showed the relatively greater investment on cancer research compared to other non-communicable diseases^(42)^. These donations reinforce the need for more transparency and policies to reduce potential conflicts of interest.

Few donations in our sample (*n* 79; 3·1 %) were earmarked for research, health and human services, environmental initiatives, scholarships and fellowships and educational initiatives. Despite the small number of donations in these categories, some of these donations were among the ten largest in monetary value in the sample (e.g. Coca-Cola’s $366 000 donations to the Hungarian Dietetic Association was classified as scholarship and fellowship). Donations that provide scholarships and other forms of financial support reflect donors’ corporate social responsibility, defined as ‘context-specific organisational actions and policies that take into account stakeholders’ expectations and the triple bottom line of economic, social and environmental performance’^(43)^. Research has shown that corporate social responsibility increases employee’s work motivation and performance^(44,45)^ and increases consumers’ loyalty and trust in the company or brand^(46,47)^. More research is needed to understand how PAO daily operations may—overtly or inadvertently—be shaped by loyalty toward their food industry donors.

The strengths of our study include the large number of donations included in the sample and our extensive data collection method. These factors allowed us to (1) examine the types of PAO that receive donations from the food industry and (2) create a comprehensive list of reasons for donations. Most previous studies on PAO focussed on donations from the pharmaceutical, device and/or biotechnology industry^(2,6,24,25)^.

Our study has several limitations. One limitation is that we did not score companies according to the percentage of their products that are unprocessed or minimally processed. It is possible that food companies that produce unprocessed foods may also engage in practices that generate conflicts of interest. Our search focussed only on tax documents, excluding other sources of donation information like PAO or food company web pages and news from reputable sources. Another limitation is the exclusion of eleven food companies due to incomplete donation data from 2000 to 2018. Finally, our study only included publicly disclosed data from 990 forms available on www.propublica.org and www.guidestar.org. It is possible, however, that these websites might not have every 990 form for each company and year in our sample. Propublica provides Internal Revenue Service data from 2013 onwards but relies on company self-reporting or investigative journalism for 2001–2012. While most years are complete, some are missing or illegible — potentially interfering with data collection. Future studies could include a complete analysis of omitted food companies to generate a more comprehensive database and could prospectively track new tax documents to reduce the probability of missing donation information that might be deleted or replaced. Lastly, future research could include other countries and international brands in order to highlight similarities and discrepancies in donation practices across different markets. Different data sources may be needed to complete an international analysis.

## Conclusions

Our study demonstrates the need for PAO to publicly disclose the receipt of donations from the food industry, as these relationships have a great impact on public health policy. Increased awareness of food industry donation practices involving PAO may generate pressure for policies mandating transparency or encourage both actors to voluntarily disclose donations. This study also provides the foundation for a comprehensive understanding of food companies’ donation practices over time. If public disclosure becomes widespread, constituents, advocates, researchers and policymakers can better supervise and address potential conflicts of interest that may arise from food and beverage company donations to non-communicable disease-focussed PAO. Ultimately, this may also allow policymakers and public health experts to enact public health policies without interference from the food industry and other corporate actors.
